# Drowning: a preventable crisis in the region

**DOI:** 10.1016/j.lansea.2025.100636

**Published:** 2025-07-10

**Authors:** The Lancet Regional Health – Southeast Asia

Each year on July 25, the world observes Drowning Prevention Day to raise awareness of the widespread yet often overlooked crisis of drowning. The WHO South-East Asia Region accounted for 28% of global drowning deaths in 2021, ranking second after the Western Pacific Region. It was also the leading cause of death due to injury among children in the region, particularly among children younger than 5 years. Drowning was the leading cause of unintentional injury-related deaths among adolescents in 2019, resulting in 45,400 deaths. According to the Global status report on drowning prevention 2024, although drowning rates declined in the region by 48% from 2000 to 2021, progress remains slow. In Bangladesh, the most affected country in the region, while drowning rates have declined by 78% since 1990, it still has the highest rate of child drowning in south Asia.

Occupational exposure, water transportation, unsafe migration journeys, and intentional drowning deaths are major contributors to drowning deaths. Children and young adults are at the highest risk of drowning. The countries in the region have extensive networks of water bodies, which serve as vital sources of livelihood—ranging from fishing to water for household chores, drinking, and transportation. However, unsafe water infrastructure, seasonal flooding, lack of swimming skills, and absence of supervision put children and young adults at high risk of drowning. Flood vulnerability during monsoon season complicates the issue because of a complete disregard of safety regulations and protective measures in the region. Socioeconomic disparities contribute significantly to drowning risk, with poorer communities having limited access to safety measures. If we do not invest today in drowning prevention, over 7.2 million people, mainly children, could die by 2050. Furthermore, fatal and non-fatal drowning incidents could generate total economic losses of about US$4 trillion in the same timeframe.

There is a clear need for strong national policies that prioritise drowning prevention. The 2024 Global status report on drowning prevention indicated that in the WHO South-East Asia region, 67% of countries have a national focal point for drowning prevention and 33% of countries have national drowning prevention strategies, both the highest among all WHO regions. However, there is still much work to be done. Effective legislation and strong enforcement of safety standards for water bodies, regulation of boating and swimming activities, and mandatory safety measures such as life jackets and fencing around water sources are crucial measures for effective drowning prevention. Policies should also promote community engagement in these programmes, as local context will play an important role in identifying key risk factors. These could include implementing early warning systems, flood barriers, and community evacuation plans to reduce flood-related drownings in flood-prone areas.

Lack of official, reliable and complete data hinder our understanding of the true scope of drowning in the region. Enhancing injury surveillance systems, integrating drowning data into national health information systems, and conducting regular community-based surveys can provide better insights into risk factors, high-risk populations, and geographical hotspots, and help with allocating resources to target populations and actions. There is a need to learn more about the cultural beliefs and strong association with water bodies that increase risks of drowning in the countries of the region.

Drowning deaths are preventable. According to WHO, drowning prevention strategies, including three basic measures, could save millions of lives: teach basic swimming and water safety skills to school-age children aged 6 years and older; provide community-based, supervised child-care for preschool children to reduce drowning risk; and train bystanders in safe rescue and resuscitation. A large-scale community intervention of sending children aged 1–3 years to creches in rural areas of Bangladesh resulted in almost 90% reduction in drowning deaths in that age group. Some research also discusses the integration of drowning prevention into broader child health and safety initiatives. Cost-effectiveness studies are now available to inform policy makers regarding resource allocation. Although we now know how we can prevent drowning and deaths related to drowning, we will need strong action from legislation and enforcement. There should be a focus on scalability and sustainability of current programmes. Now is the time to incorporate drowning prevention into broader public health goals such as disaster preparedness, climate resilience, child protection, and poverty alleviation. Emerging technologies such as mobile apps, drone surveillance, and AI-based early warning systems are severely underutilised in the regional context. The field also needs to develop evidence and action on improving emergency response, first aid training, and rehabilitation services for drowning victims. As a flood-prone country, Bangladesh introduced community-based warning systems that have helped in reducing disaster risk and improving disaster management over the past three decades.

It is time to start investing in drowning prevention strategies, particularly for the most affected communities, empower them with decision-making tools, and help them build resilience to disasters so that they can sustain their lives amidst fragile ecosystems. It is also time to build knowledge in the field through multisectoral partnerships to understand risk factors and patterns, and to develop scalable, adaptable interventions in response to the rapidly changing climate.

